# Large-scale adaptive divergence in *Boechera fecunda,* an endangered wild relative of *Arabidopsis*

**DOI:** 10.1002/ece3.1148

**Published:** 2014-07-22

**Authors:** Larry J Leamy, Cheng-Ruei Lee, Vanessa Cousins, Ibro Mujacic, Antonio J Manzaneda, Kasavajhala Prasad, Thomas Mitchell-Olds, Bao-Hua Song

**Affiliations:** 1 Department of Biological Sciences, University of North Carolina at CharlotteCharlotte, North Carolina, 28223; 2 Department of Biology, Duke UniversityDurham, North Carolina, 27705; 3 Departamento de Biología Animal, Biología Vegetal y Ecología, Universidad de JaénJaén, 23071, Spain; 4 Department of Biology, Colorado State UniversityFort Collins, Colorado, 80523

**Keywords:** Conservation, endangered species, environmental differentiation, *F*_ST_, local adaptation, *Q*_ST_

## Abstract

Many biological species are threatened with extinction because of a number of factors such as climate change and habitat loss, and their preservation depends on an accurate understanding of the extent of their genetic variability within and among populations. In this study, we assessed the genetic divergence of five quantitative traits in 10 populations of an endangered cruciferous species, *Boechera fecunda*, found in only several populations in each of two geographic regions (WEST and EAST) in southwestern Montana. We analyzed variation in quantitative traits, neutral molecular markers, and environmental factors and provided evidence that despite the restricted geographical distribution of this species, it exhibits a high level of genetic variation and regional adaptation. Conservation efforts therefore should be directed to the preservation of populations in each of these two regions without attempting transplantation between regions. Heritabilities and genetic coefficients of variation estimated from nested ANOVAs were generally high for leaf and rosette traits, although lower (and not significantly different from 0) for water-use efficiency. Measures of quantitative genetic differentiation, *Q*_ST_, were calculated for each trait from each pair of populations. For three of the five traits, these values were significantly higher between regions compared with those within regions (after adjustment for neutral genetic variation, *F*_ST_). This suggested that natural selection has played an important role in producing regional divergence in this species. Our analysis also revealed that the *B. fecunda* populations appear to be locally adapted due, at least in part, to differences in environmental conditions in the EAST and WEST regions.

## Introduction

Because of increasing habitat loss and/or fragmentation, climate change, and various other factors, many species currently are in decline and face potential extinction (Pimm et al. 1995; Kramer and Havens 2009; Stuart et al. 2012). This has long been a concern especially for those species in populations experiencing reduced effective population sizes and/or restricted gene flow (DeSalle and Amato 2004; Frankham 1995, 2005; Ouborg et al. 2006). While individuals in these populations may well be adapted to a particular local environment (Prosperi et al. 2006), their ultimate survival generally will depend on whether they possess sufficient adaptive genetic diversity to cope if changes should occur in the environment (Gienapp et al. 2008) or if they are transplanted to different environments (Montalvo and Ellstrand 2001; Grondahl and Bodil 2008) for conservation purposes.

Adaptive genetic variation in quantitative traits, especially various fitness components, is key for understanding the evolutionary potential of various species (Lande 1980; Mousseau and Roff 1987; Houle 1992; Frankham 2010). But many genetic studies of endangered and threatened species have only used neutral molecular markers such as microsatellites (Chase et al. 1996; Ellis et al. 2006; Kramer and Havens 2009; Baskauf et al. 2013). While these studies have greatly contributed to our understanding especially of the structure and extent of gene flow among populations, they have been considered less useful in assessing whether population divergence is adaptive (Storfer 1996; Kramer and Havens 2009). In recent years, evidence of adaptive divergence often has come from studies using both neutral molecular and quantitative genetic traits. Typically, this has involved comparisons of quantitative genetic divergence among populations, *Q*_ST_, with a baseline measure of neutral population divergence, *F*_ST_ (reviews in Whitlock 2008; Leinonen et al. 2013). The majority of these studies have found that *Q*_ST_ values exceed those for *F*_ST_ (McKay and Latta 2002; Leinonen et al. 2013), suggesting that differentiation of various traits among population is due to divergent selection (Lande 1992). However, *Q*_ST_–*F*_ST_ comparisons have only rarely been used to address plant conservation issues (Gravuer et al. 2005).

A number of studies have shown that environmental variables can also contribute to population differentiation (Kozak and Wiens 2006; Hubner et al. 2009). Provided natural selection has been identified as a possible cause of differences among populations, these studies are useful in identifying those environmental conditions to which individuals in these populations are locally adapted. For example, Lee and Mitchell-Olds (2011) demonstrated that water availability was the key environmental variable producing genetic differentiation (measured by *F*_ST_) between two major genetic groups of *Boechera stricta*. As in this example, most of these sorts of studies have used environmental factors and either neutral molecular or quantitative genetic traits. Here, we make use of both neutral molecular and quantitative genetic traits as well as environmental variables to understand population differentiation and conservation in *Boechera fecunda* (Brassicaceae), an endangered wild relative of *Arabidopsis*.

*Boechera fecunda* is a perennial, predominantly diploid species. It is endemic to Montana and grows in areas of relatively sparse vegetation on steep slopes with periodic natural erosion (http://fieldguide.mt.gov/detail_PDBRA06290.aspx). *Boechera fecunda* is restricted to only 21 populations in two geographical regions (WEST and EAST) in western Montana (Rollins 1993) separated by a distance of ∼100 km and is listed as a threatened species by the US Fish and Wildlife Service (USDI-FWS 1993). The altitude for populations in the WEST region (4683 feet) is much lower than those in the EAST region (6746 feet). This species long-term persistence is challenged by many factors, such as livestock grazing, spotted knapweed invasion, road construction, and mining (http://fieldguide.mt.gov/detail_PDBRA06290.aspx).

Previous analyses of neutral genetic (microsatellite and nucleotide sequence) data in these populations showed that they possess levels of genetic variability comparable to a nonendangered congener and that they exhibit considerable differentiation (*F*_ST_ = 0.57; Song and Mitchell-Olds 2007). In addition, Bayesian-based analyses on microsatellite data demonstrated that the WEST and EAST populations clustered separately, and bottleneck analysis suggested that they have experienced different evolutionary histories (Song and Mitchell-Olds 2007). In our investigation, we extend this previous analysis through the use of quantitative traits (e.g., leaf and rosette size and water-use efficiency, WUE), which are reported to be important environmentally adapted traits (Dudley 1996a,b). This allowed *F*_ST_–*Q*_ST_ regional comparisons and tests for divergent selection. We also analyzed a number of environmental variables to assess their contribution in producing differentiation among the populations. In particular, we were interested in testing for local adaptation in *B. fecunda* in the two genetically and geographically different regions, and if found, in determining whether differences in specific environmental variables might account for this divergence.

## Materials and Methods

### Plant collection

**Figure 1:**
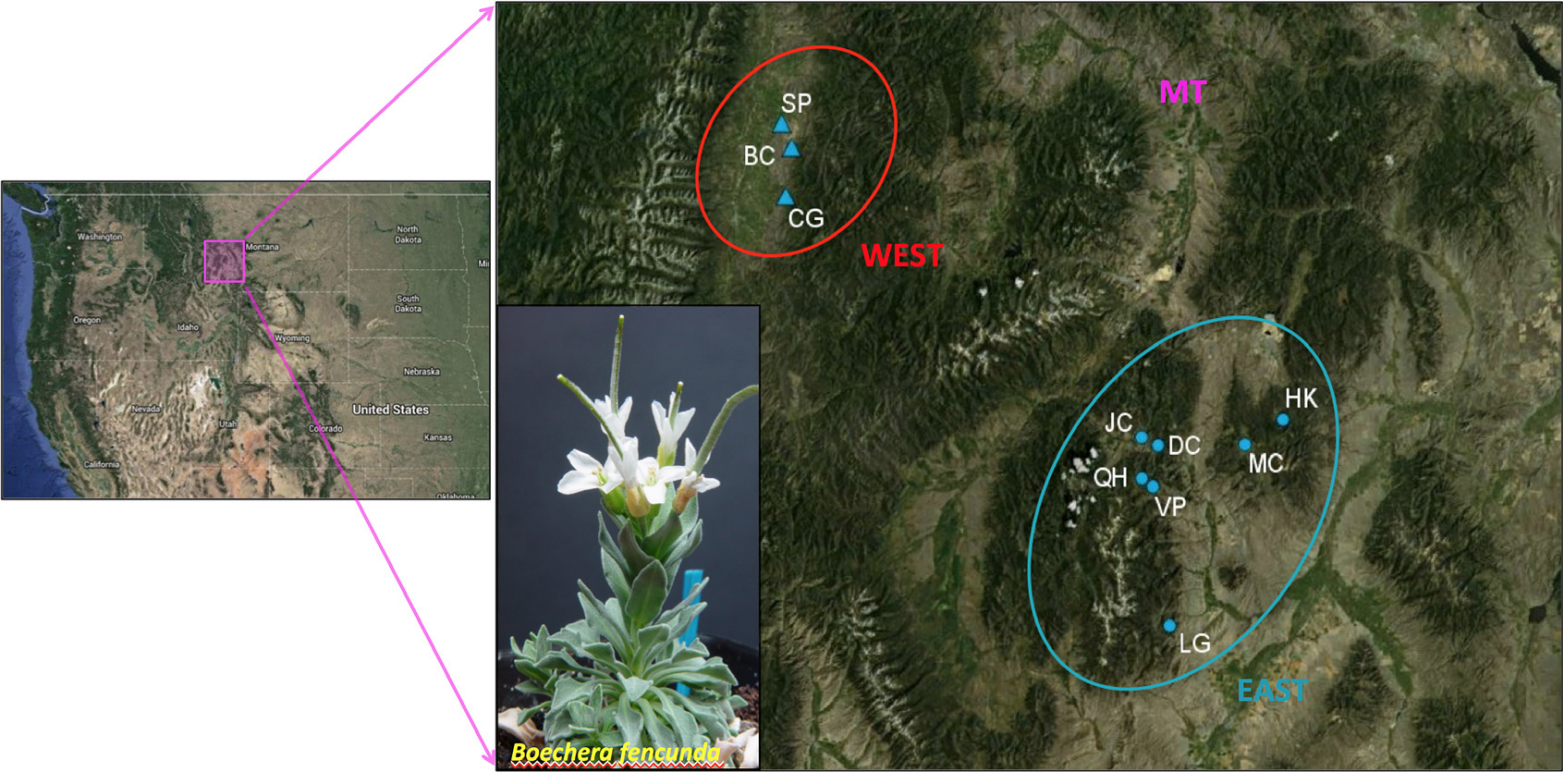
A map showing the picture of *Boechera fecunda* as well as locations of the three WEST (triangles) and seven EAST (circles) populations of *Boechera fecunda* in southwestern Montana.

Ten to twenty *B. fecunda* genotypes per population were collected from ten populations (generation *S*_0_), three in the WEST geographical region and seven in the EAST geographical region of their range in Montana (Fig. 1, Supplemental Table S1). These ten populations represent the whole species range. All plants were planted in pots and randomly arranged into flats in the Duke University greenhouse that was maintained at a 16-h day length (6 am to 10 p.m.), a daytime temperature of 18.3–21.1°C and a nighttime temperature of 12.8–15.6°C. All genotypes were propagated through self-fertilization and single seed descent for a total of two generations to reduce maternal effects. *B. fecunda* has a high self-fertilization rate in natural environments (Song and Mitchell-Olds 2007); so, selfed seeds obtained from each individual genotype were regarded as seed family replicates. Some seeds did not germinate, however; so the number of families and replicates per family varied among the 10 populations and was reduced from the maximum possible (Supplemental Tables S2 and S3).

### Measurement of quantitative traits

During the 8th week of growth of each of the seedlings from the *S*_2_ seeds, their rosette diameter (RosD) and height (RosH) were measured in cm. Rosette volume (RosV) was calculated as an additional trait by *πr*^2^*h*/3, where *r* = the radius and *h* = the height of the rosette (Lee and Mitchell-Olds 2013). In addition, we also measured (in cm) the maximum width of a young leaf (Leaf) in the top (youngest) cluster. Altogether, these traits were measured in a total of 475 plants (*N* = 472 for RosV).

During the 10th week of growth, instantaneous water-use efficiency (WUE) was measured in each plant. WUE was calculated by dividing the carbon fixation rate (A) by the water transpiration rate (E). A and E were recorded on whole plants using a modified system and protocol (Tonsor and Scheiner 2007) based on a Li-Cor LI-6400 apparatus (Li-Cor, Lincoln, NE). Each plant (total *N* = 230) was put in a separate cuvette from which three measurements were taken with a 10-s interval once the concentration of CO_2_ had stabilized. All measurements were made between 9 am to 5 pm with roughly 400 *μ*mol^−1^/mol^−1^ CO_2_ and 26% relative humidity in the surrounding environment. The mean of the three measurements was used in further analyses.

### Descriptive statistics

Prior to the quantitative genetic analysis, we first examined the distributions of each of the five (one leaf, three rosette, and WUE) traits after adjustment for differences among populations (residuals from ANOVAs with population as the factor). All were found to be normally distributed except RosV, which showed some skewness that was corrected with a logarithmic transformation. We calculated basic statistics (means and standard deviations) for each of the traits for populations in the separate EAST and WEST regions and conducted an ANOVA to test for differences in their means between these regions. We used a nested ANOVA model with regions as a fixed factor, populations as a random factor nested within regions, and families as a random factor nested within populations. Probabilities associated with the *F* tests of regional differences also were evaluated with the false discovery rate procedure, FDR (Benjamini and Hochberg 1995). We also calculated correlations among these traits and evaluated the significance of their associated probabilities generated from Student's *t*-tests again using the FDR procedure. Finally, we ran a principal component analysis to explore the covariance patterns in these traits and plotted the means of the scores of the first principal component (PC1) on the *x*-axis and the second principal component (PC2) on the *y*-axis for each of the 10 populations to discover patterns of dispersion among these populations.

### Microsatellite analysis

Molecular data for 13 polymorphic microsatellite loci were retrieved from data collected by Song and Mitchell-Olds (2007). A matrix of *F*_ST_ values (Weir and Cockerham 1984) for each of the 45 pairs of populations then was generated using GenAlex ver. 6.5 (Peakall and Smouse 2012). *R*_ST_ values (Slatkin 1995) also were calculated as they are often advocated as more appropriate for microsatellites, but results were similar for both *R*_ST_ and *F*_ST_ values, and we therefore use *F*_ST_ estimates throughout the analysis. We derived geographical distances using the latitude and longitude for each population obtained from the “fields” package (http://CRAN.R-project.org/package=fields) in R. To test for isolation by distance (IBD) among the studied populations, we used a Mantel's test of association of the *F*_ST_ values in each of the 45 population pairs with the matrix of geographical distances.

### Quantitative genetic analysis

We used the MIXED procedure in SAS to estimate variance components for four random factors in a nested ANOVA model. The variance components for the factors affecting each of the five traits were regions (*V*_*R*_), populations nested within regions (*V*_*P*_), families nested within populations (*V*_*F*_), and the error (*V*_*E*_) from replicates within families. Regions was treated as a random factor in these analyses so that its percent contribution to the total variance could be assessed. Broad-sense heritabilities for each trait were calculated by *V*_*F*_/(*V*_*F*_ + *V*_*E*_) within populations, and sampling with replacement (bootstrapping) among the families was performed with 1000 iterations to estimate 95% confidence intervals for the heritabilities. Beyond heritabilities, we also calculated the coefficient of genetic variability, CV_G_, for each trait by 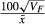, where 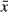 is the mean of the trait (Houle 1992). CV_G_ is a measure of how much phenotypic change may occur given a unit of selection (“evolvability”).

### *Q*_ST_–*F*_ST_ comparisons

In many previous studies, *Q*_ST_ values for various quantitative traits have been estimated and directly compared with *F*_ST_ estimates (Leinonen et al. 2013) to infer the cause of population differentiation. Because of potential *F*_ST_ outliers, Whitlock (2008) has emphasized that *Q*_ST_ values (or their mean) ideally should be compared to the distribution of *F*_ST_ values. *Q*_ST_ values exceeding an appropriate threshold (typically the 97.5 percentile) *F*_ST_ value then are taken as evidence of divergent selection. Our total of 13 molecular markers genotyped for the *B. fecunda* populations, however, is far fewer than the minimum of 50 recommended by Whitlock (2008) to ensure a reasonable description of the *F*_ST_ distribution. Alternatively, estimation of an *F*_ST_ threshold may be made from the chi-square distribution as outlined by Lewontin and Krakauer (1973). Several of the among-population *F*_ST_ values for the 13 traits were rather high (three values >0.6) in magnitude, however, and Whitlock (2008) has shown that the Lewontin–Krakauer method does not work well for *F*_ST_ values >0.1.

For these reasons, and because our primary interest was in testing for regional genetic divergence, we used an alternate approach to the traditional *Q*_ST_–*F*_ST_ direct comparisons. Specifically, we calculated *Q*_ST_ values for each of the five traits for each of the 45 pairs of populations. These values were obtained by *V*_*P*_/(*V*_*P*_ + 2*V*_*F*_), where *V*_*P*_ and *V*_*F*_ were estimated from nested ANOVAs (factors included populations, families within population, and residual within families). *V*_*F*_ estimates the genetic variance among families within populations and is equivalent to the *V*_A_ term in the formula for *Q*_ST_ shown by Whitlock and Gilbert (2012) to be appropriate when calculated from nested ANOVAs. *F*_ST_ values (Weir and Cockerham 1984) for each of the 45 pairs of populations were calculated from the 13 microsatellite loci as previously described.

To determine whether any differences between the two regions might be due to natural selection, we calculated partial correlations between the *Q*_ST_ values and regional differences, adjusting for neutral molecular variation. This was performed using a partial Mantel's procedure available in the ECODIST package in R (http://CRAN.R-project.org/package=ecodist) with a matrix of *Q*_ST_ values, a 0/1 matrix defining population pairs within and between regions, and an *F*_ST_ matrix. Probabilities for the Mantel's correlations were estimated from matrix permutations (10,000 iterations) and evaluated using the FDR procedure. Positive and statistically significant correlations between pairwise *Q*_ST_ values and the within/between region population relationship were taken to indicate that quantitative genetic differences between regions were greater than those within regions after adjustment for neutral genetic effects, and therefore that regional variation was ascribable to divergent selection. Saether et al. (2007) strongly advocated this pairwise approach for *Q*_ST_ comparisons and used it to infer local adaptation in populations of great snipe in two northern European regions.

### Environmental analysis

We also were interested in investigating the contribution of environmental factors to the differentiation of *B. fecunda* among the populations. Following Lee and Mitchell-Olds (2011), we obtained data on 26 environmental variables for each of the populations. These included the altitudes of each of the populations, 19 “Bioclim variables” obtained from WorldClim (Hijmans et al. 2005), five topographical variables (aspect, slope, flow direction, flow accumulations, and compound topographical index) obtained from the HYDRO1k database of the U.S. Geological Survey (USGS), and distances to the nearest stream measured in Google Earth. Each variable was log-transformed, a constant added so that the minimum value equaled one, and standardized to a mean of 0 and a variance of 1. We then used principal components analysis to discover the covariance patterns among these variables and plotted the first two principal components to visualize their effects in separating the 10 populations.

We also tested for potential effects of environmental variables in shaping quantitative genetic differences among populations. For this purpose, we used *Q*_ST_ values for the five traits in each of the 45 pairs of populations and environmental distances between each population pair. The environmental distances were calculated from Euclidean distances between each population pair, giving each of the environmental variables equal weight. Mantel's procedure then was used to test for associations of each of the *Q*_ST_ matrices with the environmental matrix in the same manner as previously described, and probabilities associated with the correlation coefficients were evaluated with the FDR procedure.

## Results

### Basic statistics

Basic statistics for the five traits over the pooled populations in each of the two geographical regions (WEST and EAST) are shown in Table 1 (means for families within populations are given in Supplemental Tables S2 and S3). Means for all traits except RosV in the WEST region are significantly greater (*P* < 0.05 from *F* tests in the ANOVAs) than those in the EAST region. This is especially true for water-use efficiency (WUE) for which the mean in the WEST region is approximately 50% larger than that in the EAST region. Trait variability is comparable between the two regions, although as judged by coefficients of variation (not shown), all traits, especially WUE, show high levels of variation.

**Table 1:** Basic statistics for the five traits in each of the two geographical regions

	WEST			EAST				
		
	*N*	Mean	SD	*N*	Mean	SD	*F*	*P*
Leaf	105	0.77	0.163	370	0.62	0.173	10.72	0.011*
RosD	105	6.83	2.041	370	5.24	1.684	9.84	0.014*
RosH	105	4.18	1.121	370	3.05	1.131	7.06	0.028*
RosV	105	1.05	0.279	367	0.82	0.279	4.17	0.075
WUE	63	1.34	0.650	167	0.89	0.521	23.78	0.001**

*N*, sample size; SD, standard deviation; *F*, the *F* values (all with 1,8 df), from ANOVAs testing the differences between the two regions *P*, probability of the *F* values.

* *P* < 0.05,

** *P <* 0.01.

**Figure 2:**
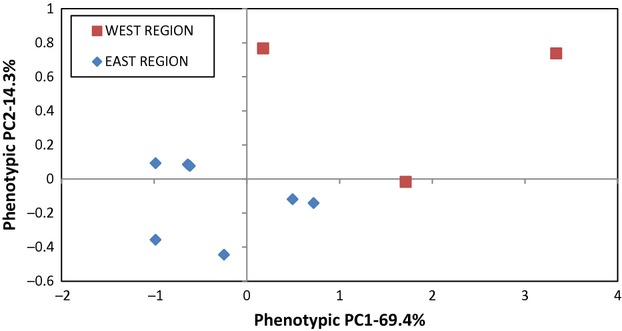
A plot of the first (PC1) and second (PC2) components from a principal components analysis of the five phenotypic traits measured in individual *Boechera fecunda* plants at the means for each of the 10 populations.

Across both pooled regions, the leaf and rosette traits show high, positive pairwise correlations (all *P* < 0.01 in Student's *t*-tests), although none of these four traits are significantly correlated with WUE (Table 2). The first two components from the principal components analysis of these correlations among the five traits explain 69.4% and 14.3%, respectively, of the total variance in these traits. Component 1 shows positive loadings on all traits except WUE and thus is a leaf–rosette component, whereas WUE dominates the second component (Table 2). The means of the scores of PC1 and PC2 for each of the 10 populations are plotted in Fig. 2. This figure shows a clear differentiation of the populations between these regions.

**Table 2:** Correlations and principal components results for the five phenotypic trails

	PCI	PCII	RosD	RosH	RosV	WUE
Leaf	0.41	−0.03	0.78	0.71	0.74	−0.06
RosD	0.41	0.02		0.74	0.98	−0.04
RosII	0.37	0.06			0.72	−0.00
RosV	0.41	0.04				−0.02
WUE	−0.02	1.00				

Shown are pairwise correlations of the five traits (all except those involving WUE are statistically significant, *P* < 0.01) measured in individual plants, and loadings for the first two components (PCI and PCII) from a principal components analysis of these correlations.

### Components of variance

Table 3 summarizes the components of variance estimated from the nested ANOVA model for each of the five traits. Within regions, family differences account for the greatest amount of variation (mean = 35.9%) for the four leaf–rosette traits, although contribute only 7% to the total variation in WUE. Regional differences are consistent among the five traits, averaging a 22.6% contribution to their total variation. Population differences contribute an average of only 8.6% of the variation, although that for rosette height (21.8%) is much larger than that for the other traits. Error contributions tend to be fairly high (mean contribution of 32.9%), especially for WUE where they account for almost 70% of the total variation. Variance components for the traits in the separate WEST and EAST regions show similar trends for differences among populations and families, although RosV and RosD exhibit higher contributions among populations in the WEST compared with the EAST region.

**Table 3:** Variance components and heritabilities for the five traits

	Variance components					
			
	Region	Population	Family	Error	Heritability	CV_G_
BOTH REGIONS						
Leaf	0.009 (24.3)	0.002 (3.9)	0.017 (43.7)	0.011 (28.1)	0.61 (0.45–0.68)	20.0
RosD	0.986 (23.6)	0.409 (9.8)	1.879 (45.1)	0.899 (21.5)	0.67 (0.53–0.74)	24.5
RosH	0.359 (20.8)	0.378 (21.8)	0.575 (33.2)	0.420 (24.2)	0.58 (0.43–0.64)	23.0
RosV	0.020 (20.5)	0.007 (7.2)	0.050 (50.6)	0.021 (21.7)	0.70 (0.58–0.77)	25.7
WUE	0.097 (23.6)	0.001 (0.3)	0.029 (7.0)	0.283 (69.1)	0.09 (0.00–0.21)	16.8
WEST REGION						
Leaf	–	0.001 (4.3)	0.019 (70.2)	0.007 (25.5)	0.74 (0.38–0.82)	17.9
RosD	–	1.217 (25.9)	2.429 (51.7)	1.054 (22.4)	0.70 (0.37–0.82)	22.8
RosH	–	0.038 (26.2)	0.546 (38.2)	0.509 (35.6)	0.52. (0.21–0.62)	17.7
RosV	–	0.018 (20.4)	0.049 (55.9)	0.021 (23.7)	0.70 (0.36–0.82)	21.0
WUE	–	0.009 (2.1)	0.075 (17.7)	0.341 (80.3)	0.18 (0.00–0.42)	20.4
EAST REGION						
Leaf	–	0.002 (5.3)	0.016 (54.6)	0.012 (40.1)	0.58 (0.08–0.74)	20.7
RosD	–	0.211 (7.6)	1.709 (61.6)	0.852 (30.8)	0.67 (0.15–0.78)	24.9
RosH	–	0.373 (27.6)	0.583 (43.1)	0.395 (29.3)	0.60 (0.17–0.69)	25.0
RosV	–	0.005 (6.3)	0.051 (61.7)	0.027 (32.0)	0.66 (0.39–0.81)	27.2
WUE	–	0.0 (0.0)	0.007 (2.6)	0.264 (97.4)	0.03 (0.00–0.42)	9.4

Shown are components of variance for regions, population, families, and error for both regions and for the two separate (WEST and eAST) regions for each or the five traits (with their percentage contributions in parentheses). Also given are heritabilities and their 95% confidence intervals (in parentheses) and coefficients of genetic variation (CV_G_) for each trait.

Within populations, heritabilities estimated from the proportion of family variance over that for family plus error variance average a moderately high 0.53 for the five traits (Table 3). The leaf and rosette traits all have heritabilities that are 0.58 or greater, and their 95% confidence intervals estimated from bootstrapping show that they are significantly different from 0. The heritability for WUE is quite low (0.09), however, and is not significantly different from 0. Heritability estimates for the leaf and rosette traits in the WEST region generally tend to be higher than those in the EAST region, although given their confidence intervals, none of these differences is statistically significant. The heritability for WUE in the WEST region (0.18) is higher than that in the EAST region (0.03), although it is still not significantly different from 0. For all populations, coefficients of genetic variation (CV_G_s) for all five traits are high (average of 22.0), with WUE having the lowest value. CV_G_ values for the leaf/rosette traits in the EAST region (mean = 23.3) exceed those for the WEST region (mean = 19.9), whereas WUE exhibits the reverse trend.

### *F*_ST_–*Q*_ST_ comparisons within and between regions

*F*_ST_ values estimated over 13 microsatellite loci for each of the 45 pairwise combinations of populations (Supplemental Table S4) vary from 0.087 to 0.849. The median values for these estimates within (0.405) and between regions (0.369) are quite similar. *Q*_ST_ estimates for the five traits also varied considerably among the 45 pairs of populations. Median values for these *Q*_ST_ values for population pairs between regions are greater than those within regions for all traits, including for RosD, RosV, and WUE where the median within regions is 0.

**Figure 3:**
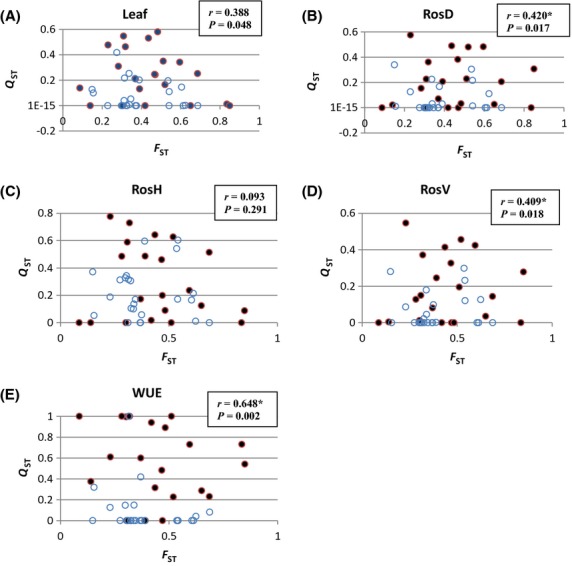
Scatterplots of *Q*_ST_ versus *F*_ST_ values for leaf (A), RosD (B), RosH (C), RosV (D), and WUE (E) in each of the 45 pairs of populations. Closed circles = population pairs between regions, open circles = population pairs within regions. The Mantel's correlation (*r*) of *Q*_ST_ and within/between region values and its associated probability is given in each case. *P < 0.05.

We tested whether the *Q*_ST_ values differed for population pairs within and between the WEST and EAST regions, after adjustment for neutral genetic variation (*F*_ST_ values), with the use of partial Mantel's tests as described earlier. Because of the non-normal distributions of the *Q*_ST_ values, we used nonparametric Spearman rather than Pearson's correlations in the ECODIST package. These correlations between *Q*_ST_ values and the between/within region index values were significantly different (*P* < 0.05) for Leaf, RosD, RosV, and WUE. Figure 3 plots the *Q*_ST_ versus *F*_ST_ values for each trait and clearly illustrates that the *Q*_ST_ values between regions predominantly are greater than those within regions.

### Environmental effects

Results of the principal components analysis of the 26 environmental variables are shown in Supplemental Table S5. The first principal component accounts for 38.6% of the total variance, and primarily represents a contrast of the temperature variables (BIO1-BIO11, mean loadings = 0.23) with the precipitation variables (BIO12-BIO19, mean loadings = 0.17). The second principal component contributes 28.1% of the total variation and loads most heavily on the precipitation variables (mean loadings = 0.26) more so than the temperature variables (mean loadings = 0.14), but also with some contributions from other variables, especially altitude and flow direction. Principal component three contributes considerably less variation (10.6%) than either of the first two components, but represents an interesting contrast between BIO2, SLOPE and STREAM with BIO12 and especially ASPECT. PC4 contributes slightly <8% of the total variation and loads most heavily on CPI, with some contrast from SLOPE.

**Figure 4:**
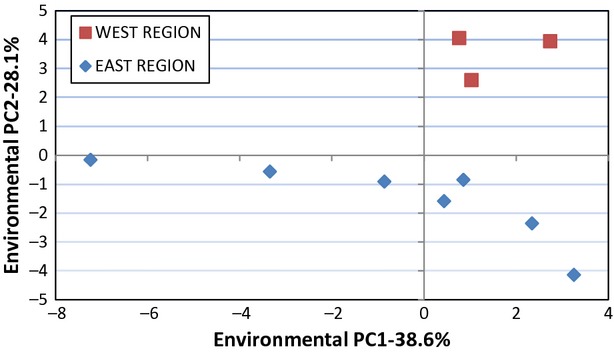
A plot of the first (PC1) and second (PC2) components for each of the 10 populations derived from a principal components analysis of 26 environmental variables.

Figure 4 shows a plot of the scores of PC1 versus PC2 for each of the 10 populations derived from the principal components analysis of the environment variables. In this plot, the three WEST populations are clustered, with the seven EAST populations showing more dispersion. PC1 for the environmental variables primarily serves to separate the seven EAST populations, whereas PC2 clearly separates all the EAST populations from the WEST populations.

Mantel's tests showed that environmental distances between pairs of populations were significantly associated with geographic distances, although not with regional differences (Table 4). Environmental differences between populations calculated from the second principal component of the environmental variables, however, were positively associated with both geographic distances and regional differences. Of the *Q*_ST_ values for the five traits, none were significantly associated with the environmental distances, but *Q*_ST_ values for three (RosD, RosV, and WUE) of the five traits were significantly correlated with the environmental PC2 distances.

**Table 4:** Mantel tests of association of environmental distances with distance, region, and *Q*_ST_ variables

First variable	Second variable	*r*	*P*
Distance	Environment	0.402	0.015*
Distance	EnvironmentPC2	0.486	0.004*
Region	Environment	0.346	0.058
Region	EnvironmentPC2	0.394	0.033*
*Q*_ST_-Leaf	Environment	−0.126	0.741
*Q*_ST_-RosD	Environment	0.129	0.277
*Q*_ST_-RosH	Environment	−0.235	0.855
*Q*_ST_-RosV	Environment	0.138	0.268
*Q*_ST_-WUE	Environment	0.297	0.058
*Q*_ST_-Leaf	EnvironmentPC2	0.328	0.040
*Q*_ST_-RosD	EnvironmentPC2	0.442	0.015*
*Q*_ST_-RosH	EnvironmentPC2	0.019	0.432
*Q*_ST_-RosV	EnvironmentPC2	0.407	0.021*
*Q*_ST_-WUE	EnvironmentPC2	0.509	0.006*

Shown are correlations (*r*) and their probabilities (*P*) from mantel tests of association of environmental distances (including distances calculated from the second principal component of an environmental variable PCA) with geographic distances, with regional distances, and with *Q*_ST_ values for each of the quantitative traits.

* *P* < 0.05.

## Discussion

Optimal preservation strategies for endangered and threatened species are based on information about the extent of genetic variability within and between populations, and the forces (e.g., selection and drift) driving population differentiation. In general, the goal is to preserve adaptive genetic variability across the range of the species (Crandall et al. 2000), especially in ecologically relevant traits related to overall fitness (Hedrick 2001; Reed and Frankham 2001; Hansson and Richardson 2005). With the development of molecular markers during the past decade, many estimates of molecular genetic diversity have been made. So far, however, very few studies have estimated quantitative genetic variation in threatened and endangered species (Frankham 2010). We undertook this study to evaluate quantitative genetic variation and to discover whether divergence selection played an important role in the differentiation between WEST and EAST populations of *B. fecunda* previously uncovered in a study using neutral genetic markers (Song and Mitchell-Olds 2007). We tested this by comparing *Q*_ST_ values for population pairs within and between the two regions while controlling for neutral genetic divergence (*F*_ST_). Further, we conducted an environmental analysis to quantify environmental differences and to discover what environmental variables, if any, might have shaped differences in *B. fecunda* between the two regions.

Our analysis of the *B. fecunda* populations showed that there appears to be ample quantitative genetic variability and potential for evolvability, especially compared with various other endangered species. For example, Benscoter et al. (2013) compared vulnerability, adaptive capacity, and ecological traits for 12 threatened and endangered subspecies to nonlisted subspecies of the same parent species, and they found that the former showed higher vulnerability and lower adaptive capacity. Also, Ye et al. (2014) measured five quantitative traits in the endangered species *Psilopeganum sinense* and found little genetic variation.

From the *Q*_ST_-region comparisons, we provided evidence for divergent selection operating to differentiate populations in the EAST from those in the WEST region. We also showed that environmental conditions may have contributed to differences between these regions in which this species has become locally adapted. This is consistent with a previous study (McKay et al. 2001) showing adaptation of *B. fecunda* to drought conditions in the WEST region. Fang et al. (2013) also found divergent selection and local adaptation in an endangered conifer species with disjunct populations. In general, our results support the previous recommendations of Song and Mitchell-Olds (2007) and McKay et al. (2001) that management efforts for *B. fecunda* should be directed to the preservation of populations in each of these two regions and in no case should transplantation of plants be attempted between the regions.

### The quantitative traits

Consistent with local adaptation expectations, the quantitative traits we measured in the *B. fecunda* populations exhibited clear differences between the two regions, with individuals in the WEST region showing significantly larger mean values in all cases. This pattern is consistent with previous measurements with smaller sample sizes for this species (McKay et al. 2001).

A well-accepted hypothesis is that selection favors higher water-use efficiency and smaller leaf size in dry compared with wet habitats (Dudley 1996a,b). In short-lived species, it is suggested that water stress, together with other environmental stress factors, can accelerate development (e.g., Stanton et al. 2000). The accelerated development may be associated with lower water-use efficiency (e.g. Stanton et al. 2000; Arntz and Delph 2001).

In our species, we found both accelerated development and higher water-use efficiency in plants in the drier (WEST) region compared with the wet region (EAST), as did McKay et al. (2001). Perhaps annual plants and perennials that go dormant may escape water limitation with a resource acquisition rather than a conservation strategy. Such a strategy would accelerate development that permits completion of reproduction before water limitation occurs (e.g., Brouillette et al. 2014). The WEST populations of *B. fecunda* have a larger leaf and rosette size, as well as an earlier flowering time (Song, pers. obs.) compared with plants in the EAST populations and may well use a resource acquisitive strategy. The higher WUE level in the WEST populations is not consistent with the accelerated development hypothesis, but is consistent with the general trend that WUE tends to increase along gradients of declining moisture.

Thus the relationship between WUE and water availability is not clear in our species. Lee and Mitchell-Olds (2013) discovered that WUE levels were not significantly different between subspecies of *Boechera stricta* living in two separate regions with divergent levels of water availability. Donovan and Ehleringer (1994) did not find evidence of selection for higher water-use efficiency in a desert shrub. No evidence of adaptive differentiation in WUE (*Q*_ST_ < *F*_ST_) was found in *Helianthus anomalus* (Brouillette et al. 2014).

It was not surprising that correlations among the leaf and rosette traits were positive and generally high in magnitude; presumably, this simply reflects overall growth processes commonly affecting these traits. Some leaf dimensions, especially leaf area per dry weight, have been found to be associated with WUE (Hoffmann et al. 2005), but none of the correlations of WUE with any of our four leaf and rosette traits were statistically significant. This independence of WUE from the leaf/rosette traits also was clearly reflected in the principal components analysis results where PC1 had positive, moderate loadings for the four leaf/rosette traits and PC2 had a positive, high loading only for WUE. Leaf/rosette trait (PC1) differences separated two (BC and SP) and WUE differences one (CG) of the three WEST populations from the seven EAST populations; so, both morphological (leaf/rosette) and physiological (WUE) traits were necessary to describe this regional divergence.

### Genetic variation in the quantitative traits

As judged by the magnitude of the heritabilities estimated from differences among families within populations in the nested ANOVAs, the leaf and rosette dimensions we measured exhibited high levels of genetic variation. Coefficients of genetic variation for these four morphological traits were even more impressive, averaging 22 over both regions. By way of comparison, CV_G_ values for leaf mass and stem length among 10 different populations of *Arabidopsis thaliana* in northern Europe averaged a considerably lower 6.9 (Stenoien et al. 2005), although that of 23.1 calculated for two measures of stem length among 10 populations of *Medicago laciniata* (Badri et al. 2008) was quite comparable.

This high level of quantitative genetic variation in the *B. fecunda* populations seems surprising for an endangered species, but the heritability estimates may have been inflated to some extent because of the decrease in within-family variation during the two generations of selfing. On the other hand, our results are compatible with those from a previous study that assessed variability in these populations from microsatellites and single-copy nuclear loci (Song and Mitchell-Olds 2007). Further, the heritability of WUE was quite low (0.09). A close species, *Boechera stricta*, also showed a low heritability for WUE (Lee and Mitchell-Olds 2013). The low heritability of WUE is not surprising because it presumably has been the target of strong selection and also possesses a high level of plasticity (Geber and Griffen 2003).

Song and Mitchell-Olds (2007) previously discovered greater neutral genetic variability in the WEST compared with the EAST populations, a trend reflected in our heritability estimates for all five traits as well (Table 3). CV_G_ values for the leaf/rosette traits, however, showed the opposite pattern, being higher in the EAST than in the WEST region. This is mainly ascribable to their lower means in the EAST region, however, because the genetic (family) variances in these four morphological traits were comparable between the two regions (Wilcoxen's signed rank test, *P* = 0.47). For WUE, the CV_G_ of 20.4 for the WEST populations was much higher than that of 9.4 for the EAST populations. More traits will be measured in the near future to fully understand patterns of quantitative genetic variation for *Boechera fecunda* in each population and region.

### Population and regional genetic divergence

Beyond family differences within populations, our analysis also provided evidence of population and regional divergence in the quantitative traits. Regional divergence was apparent from the significant differences in the means of four of the five traits in the WEST versus EAST regions, from the clear separation of the populations in these two regions in the principal components analysis of the phenotypic correlations of the traits and from the nested ANOVAs where regional differences for each trait contributed on average about 23% of the total variation. Divergence among populations within regions was much less (mean contribution = 9%). If we had analyzed divergence only among the 10 populations, the mean contribution would have increased to 20%, but we would have missed the heavy contribution of regional differences to this divergence. Clearly, it is important to understand the spatial scales on which divergence occurs (Volis et al. 2005).

In the *Q*_ST_ analysis, we tested each of the five traits separately in part to discover whether the results for WUE, a physiological trait, might differ from those for the four leaf/rosette morphological traits. And in fact, WUE showed the strongest Mantel's correlation with regional differences in these tests (see Table 4), suggesting it is most strongly affected by divergent selection. Further, Mantel's tests showed no significant association of the *F*_ST_ values and geographic distances for each pair of populations (Spearman *r* = 0.215; *P* = 0.13). This implies that genetic drift alone cannot explain the regional genetic differentiation, and that is most likely ascribable to the action of divergent selection.

### Environmental influences

We used a suite of 26 environmental variables to probe what conditions in the EAST versus WEST regions may have played a role in shaping local adaptations in the *B. fecunda* populations. These variables are not exhaustive, and other environmental measures may be important, but they do represent a good sampling especially of temperature and precipitation measures. The principal components analysis of these environmental variables showed that the first two components were effective in separating the 10 populations. PC2 was particularly interesting, because it separated populations in the two regions. This component was a complex contrast, with high loadings especially on most of the precipitation variables, but with contributions from other variables. It was not surprising that a Mantel's test showed a significant association of environmental and geographic distances, because many of the environmental variables vary considerably among the populations, especially as the distance increases. More interesting was the finding that the *Q*_ST_ values showed no association with the environmental distances for the population pairs, but three of the five *Q*_ST_ values exhibited significant correlations with the environmental PC2 distances. These results suggest that the variables described by the second environmental principal component may be contributors to local adaptation of *B. fecunda* populations in the EAST and WEST regions.

### Summary

In summary, we used quantitative genetic, neutral genetic and environmental data to analyze diversity and ecological adaptation in *B. fecunda*. For this nonmodel species, next generation sequencing technology that is now available will allow genome-wide genotyping at reduced costs and thus provide a greater range of molecular markers from which to calculate *F*_ST_ distributions. Combined with additional phenotypic traits, this should give us a more detailed understanding of population divergence and ecological adaptation in this species and of the genetic basis of ecologically important complex trait variation as well.

## Supporting Information

**Table S1.** Description and sample size of 10 natural population of *Boechera fecunda*. **Table S2.** Sample size and means for the four leaf/rosette traits in each of the 10 populations (number in bracket are the mean number of individuals per family). **Table S3.** Sample sizes and means for WUE in each of the 10 populations. **Table S4.** Pairwise population Fst values calculated from 13 microsatallite markers. **Table S5.** Principal components analysis of the environmental variables.
