# Supplementary figures and images for: Large-scale adaptive divergence in Boechera fecunda, an endangered wild relative of Arabidopsis
Source: Ecol Evol. 2014 Jul 22;4(16):3175–86. doi: 10.1002/ece3.1148 (PMC4222205; doi:10.1002/ece3.1148)

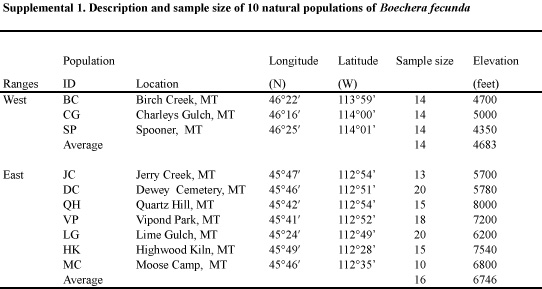


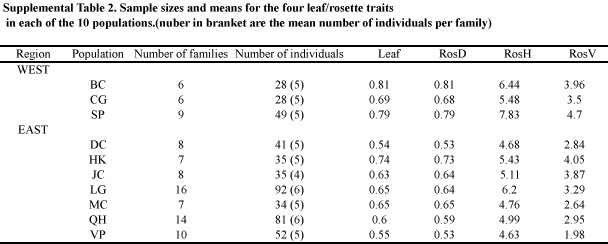


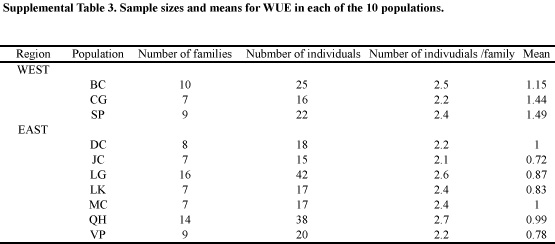


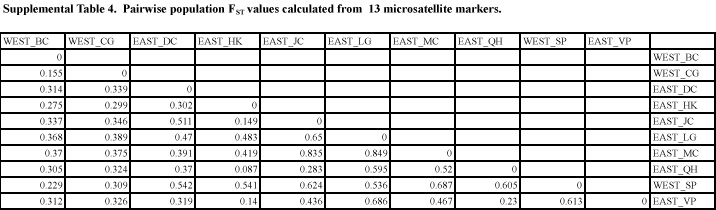


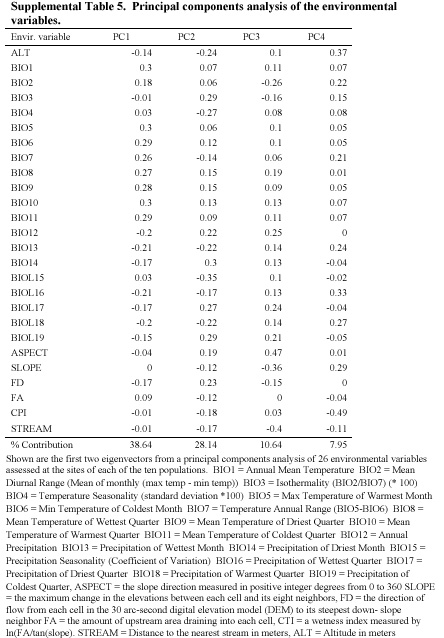

Supplement: Supplementary file 1 [file ece30004-3175-sd1.docx]
